# Botulinum Toxin for Central Neuropathic Pain

**DOI:** 10.3390/toxins10060224

**Published:** 2018-06-01

**Authors:** Jihye Park, Myung Eun Chung

**Affiliations:** 1 Department of Rehabilitation Medicine, Seoul St. Mary’s Hospital, College of Medicine, The Catholic University of Korea, 222, Banpo-daero, Seocho-gu, Seoul 06591, Korea; sophia@catholic.ac.kr; 2 Department of Rehabilitation Medicine, St. Paul’s Hospital, College of Medicine, The Catholic University of Korea, Wangsan-ro 180, Dongdaemoon-Gu, Seoul 02559, Korea

**Keywords:** botulinum toxin, BTX, central neuropathic pain, spinal cord injury, post-stroke shoulder pain, complex regional pain syndrome

## Abstract

Botulinum toxin (BTX) is widely used to treat muscle spasticity by acting on motor neurons. Recently, studies of the effects of BTX on sensory nerves have been reported and several studies have been conducted to evaluate its effects on peripheral and central neuropathic pain. Central neuropathic pain includes spinal cord injury-related neuropathic pain, post-stroke shoulder pain, multiple sclerosis-related pain, and complex regional pain syndrome. This article reviews the mechanism of central neuropathic pain and assesses the effect of BTX on central neuropathic pain.

## 1. Introduction

Botulinum toxins (BTXs) are neurotoxic substances produced by *Clostridium botulinum*, a gram-positive anaerobic bacterium. In botulism poisoning, flaccid paralysis occurs by inhibiting the release of neurotransmitters from the peripheral cholinergic nerve terminals of the skeletal and autonomic nervous system. Paralysis begins at the ocular muscles and then spreads to the muscles of the face, before reaching the respiratory muscles and causing respiratory failure.

BTX has traditionally been found in seven serotypes: A, B, C1, D, E, F, and G [1]. They have similar molecular weights and common subunit structures, but differ in their reaction mechanisms, durations of effect, and side effects.

In recent years, using molecular genetic analysis, many genes have been discovered that encode new BTXs. Thus, subtypes, such as BTX/A1, BTX/A2, BTX/B1, and BTX/B2, and chimeric types, such as BTX/DC, BTX/CD and BTX/FA, have been found.

Clinical use of BTX began in 1973, when Scott demonstrated that injecting the toxin into orbicularis oculi muscles was effective in treating strabismus. Over the next several decades, its application expanded to a variety of diseases.

BTX has a molecular weight of 150 kDa, consists of an inactive single-chain polypeptide, and folds into a 3-domain structure. The light chain (50 kDa) is a zinc-dependent protease that constitutes an active toxin and cleaves the soluble *N*-ethyl-maleimide-sensitive factor attachment receptor (SNARE) complex [2,3]. The heavy chain (100 kDa) consists of an N-terminal translocation domain and a C-terminal receptor binding domain, and it acts in neuron-specific binding. The light and heavy chains are linked by disulfide bonds, which partially obscure the active sites of the toxin. When the single-chain disulfide bond is reduced, the light chain metalloprotease can be released to act as a toxin.

The main functional effect of BTX occurs in the neuromuscular junction, where it inhibits the release of acetylcholine from the presynaptic nerve ending, resulting in muscular and autonomic paralysis [4]. The toxin-mediated muscle relaxation process proceeds in three phases: Binding, internalization, and the inhibition of neurotransmitter release. Specific binding to neurons is mediated by heavy chains [5] and internalization is mediated by receptor-mediated endocytosis [6,7]. Once BTX is internalized, the light chains within the vesicles are translocated across the vesicle membrane and released into the neuronal cytoplasm. SNARE is involved in the exocytosis of acetylcholine vesicles, located at the nerve endings, by attaching acetylcholine vesicles to the cell membrane, thus, allowing acetylcholine exocytosis to occur [8]. BTX causes degradation of the SNARE protein, resulting in paralysis. Based on these mechanisms, BTX is clinically used to treat muscle spasticity associated with central nervous system (CNS) disorders, such as stroke, brain injury, spinal cord injury (SCI), cerebral palsy, and multiple sclerosis (MS).

However, two other functional effects of BTXs exist: The effects on the afferent limb of the motor nervous system and the analgesic effect on the sensory nerve system. Several preclinical studies have shown that BTX inhibits neuromodulator and transmitter secretion, which is important for neurotransmission in the sensory pathway, and, thus, BTX may reduce neuropathic pain.

Preclinical and clinical studies have reported the effects of BTX on peripheral neuropathic pain and, generally, demonstrated a high level of evidence for some diseases [9]. However, few studies have reported its therapeutic effects on central neuropathic pain, and its effects have not been proven. The aim of this article is to review the mechanism of central neuropathic pain and to investigate the effect of BTX on central neuropathic pain.

A PubMed and EMBASE search (1980~March 2018) was performed as follows: ‘Botulinum toxins’, ‘neuropathic pain’, ‘neuropathy’, ‘pain’, ‘allodynia’, ‘hyperalgesia’ and ‘spinal cord injury’, ‘post-stroke pain’, ‘multiple sclerosis’, and ‘complex regional pain syndrome’. The results included animal studies, randomized controlled trials (RCTs), observational studies, case reports, and reviews. Editorials, guidelines, and trial protocols were excluded. Two reviewers individually assessed the abstracts to determine the eligibility of the studies. Articles not available in English and studies conducted in children (≤18 years of age) were also excluded.

## 2. Mechanism of Central Neuropathic Pain

The International Association for the Study of Pain (IASP) defines neuropathic pain as pain caused by a lesion or disease of the somatosensory nervous system [10]. Neuropathic pain is a clinical description that requires a demonstrable lesion or a disease that satisfies the established neurological diagnostic criteria. It has two typical symptoms, allodynia and hyperalgesia. Allodynia describes a pain due to a stimulus that does not normally provoke pain and hyperalgesia refers to increased pain from a stimulus that normally provokes pain [10].

Several molecular mechanisms are involved in the development of allodynia and hyperalgesia. After nerve injury, changes in the expression of sodium and calcium channels cause spontaneous activity in nerve endings, resulting in spontaneous pain. This is an important factor that causes sensitization. In addition, various cytokines, including glutamate, substance P, and proinflammatory cytokines, are involved in sensitization. Inflamed or ischemic tissues become acidified and this cellular environment causes pain by stimulating the release of neuropeptides from the primary afferent nerve tissue [11]. When neuropeptides, such as calcitonin gene-related protein (CGRP) and substance P, are secreted into the endoneurium, they cause local blood flow and blood vessel leakage, leading to edema and pain.

Because central neuropathic pain is defined by IASP as a pain caused by a lesion or disease of the central somatosensory nervous system [10], central neuropathic pain is a heterogenous group of neuropathic pain conditions. Major diagnostic conditions include: (1) Central pain associated with SCI; (2) central post-stroke pain; and (3) central pain associated with MS.

**Figure 1:**
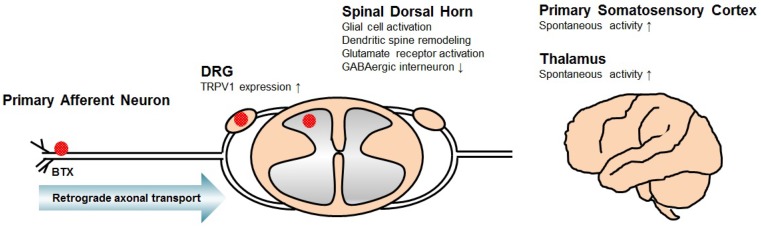
Illustrated mechanism of central neuropathic pain associated with spinal cord injury (SCI). These mechanisms include transient receptor potential vanilloid type 1 (TRPV1) overexpression in dorsal root ganglion (DRG) neurons, glial cell activation, dendritic spine remodeling, glutamate receptor activation and loss of GABAergic interneuron in the spinal dorsal horn, and spontaneous firing of neurons in the thalamus and primary somatosensory cortex. It has been suggested that the antinociceptive mechanism of botulinum toxins (BTXs) applied to the nerve endings not only affects the primary afferent neurons but also acts on the DRG and spinal dorsal horn through retrograde axonal transport.

The mechanisms of neuropathic pain, following SCI, in various animal models have been published (Figure 1). Cellular and molecular responses to SCI occur at various levels, from the distal terminals of primary afferent neurons to the cortical area along the nervous system, leading to neuropathic pain.

Several changes in the primary afferent neuron have been suggested. A definite factor that mediates SCI-related pain is an increased excitability of dorsal root ganglion (DRG) neurons. The spontaneous activity of nociceptive DRG neurons has been shown to increase after SCI [12], and some authors have demonstrated that the expression of the capsaicin-sensitive cation channel transient receptor potential vanilloid type 1 (TRPV1) in DRG neurons increases after SCI, which is enhanced by capsaicin-evoked ion currents and calcium responses in DRG neurons [13].

Neuronal hyperexcitability in the spinal dorsal horn is also associated with SCI-related neuropathic pain [14,15]. This phenomenon might occur through chronic glial cell activation [16,17], dendritic spine remodeling [18,19], dysregulation of glutamate homeostasis [16], glutamate receptor activation [20], loss of GABAergic inhibitory interneurons [21,22], interruption of descending inhibitory modulation by serotonin [23,24], or upregulation of voltage-gated calcium channel alpha-2-delta-1 subunit proteins [25].

In patients with central pain following SCI, neurons in the somatosensory thalamus fire in bursts of action potentials more frequently than do similar neurons in patients without pain [26]. In rats with contusive SCI, thalamic ventralis postero-lateralis neurons exhibited a dysrhythmia in that a significantly higher proportion fired spontaneously when compared with neurons in uninjured rats [27]. Based on these results, abnormal thalamic processes following SCI may mediate neuropathic pain. Additionally, in rats with SCI, neurons in the primary somatosensory cortex had significantly higher spontaneous firing rates, greater evoked responses to noxious mechanical stimulation, and a greater tendency to fire bursts of action potentials [28]. Another study also revealed that phosphorylation of AMPA-type glutamate receptors in the primary somatosensory cortex play an important role in the development of hypersensitivity after SCI [29].

The pathophysiology of central post-stroke pain, another important disease constituting central neuropathic pain, remains uncertain. Recent advances in brain imaging technology have increased the understanding of the role of specific anatomic locations. Several reports have suggested that central post-stroke pain commonly occurs in lesions affecting the thalamus, parietal cortex, dorsal putamen, posterior internal capsule, dorsal basal ganglia, brainstem, and lateral medulla [30,31,32,33]. Particularly, spinothalamic tracts terminated with the ventral posterolateral thalamus and lesions of the ventral posteromedial thalamus and medial lemniscal thalamocortical pathway were found to be the major factors causing central post-stroke pain [34,35]. These results have also been demonstrated in animal model experiments. Thermal hypersensitivity was observed in a rat model of the ventral posterior thalamic infarction [36], with thermal and mechanical hyperalgesia developing in rats with a thalamic hemorrhagic lesion [37].

In the middle cerebral artery occlusion rat model, increased *N*-methyl-d-aspartate (NMDA) and AMPA receptor mediated excitatory transmission of the dorsal horn, decreases in GABA and glycine receptor mediated inhibitory transmission, and an increase in descending facilitation was proposed to be involved in the development of central post-stroke pain [38]. In the ventral posterior thalamic lesion rat model, P2X7 expression in the medial thalamus was directly involved in nociceptive transmission, and short-term P2X7 inhibition led to a reduction of glutamatergic facilitation and neuronal hyperexcitability [39].

## 3. Mechanism of BTX for Central Neuropathic Pain

The initial analgesic effect of BTX is caused by a decrease in muscle spasms. However, many preclinical and clinical studies suggest that a different mechanism underlies the analgesic effect of BTX. The hypothesis is that BTX inhibits the secretion of neuropeptides and suppresses inflammation and pain.

Several preclinical studies have shown that BTX-A inhibits the release of neurotransmitters that regulate pain and inflammation. McMahon et al. showed that BTX preferentially attenuates the slow phase of KCl-evoked glutamate release, which may be associated with synaptic vesicle mobilization according to a study that utilized a guinea pig formalin-induced pain model [40]. Welch et al. reported that BTX inhibits potassium-evoked substance P secretion from cultured embryonic rat DRG neurons [41], and Durham et al. demonstrated that BTX-A can directly decrease the release of CGRP from cultured rat trigeminal ganglion neurons [42]. 

Xiao et al. demonstrated that BTX significantly reduces TRPV1 expression [43]. A neuropathic pain model was induced by transection of the lumbar 5 ventral root in male rats. BTX-A or normal saline was administered to the plantar surface by subcutaneous injection. TRPV1 expression increased significantly in the lumbar 4–5 DRG after the transection of the lumbar 5 ventral root, and this increase persisted for at least 21 days. Subcutaneous injection of BTX-A significantly, and dose-dependently, reduced the expression of TRPV1 in the DRG neuron and significantly reduced hyperalgesia. A similar effect occurred on the expression of P2X3, one of the purinergic receptors associated with nociceptors, in a study that evaluated the effect of BTX on P_2_X_3_ expression, with the same method. Subcutaneous administration of BTX-A significantly, and bilaterally, reduced mechanical allodynia and inhibited the P_2_X_3_ overexpression induced by the transection of the lumbar 5 ventral root [44]. 

One possible interpretation of these findings is that BTX reduces peripheral sensitization and afferent input to the spinal cord by inhibiting the release of neurotransmitters from peripheral nerve endings, thereby, indirectly a decreasing central sensitization. However, it has been hypothesized that the central effect may be direct by retrograde axonal transport of BTX along the branches of nociceptive neurons.

Immunohistochemical experiments have revealed that cleaved SNAP-25, a product of BTX-A action, is found not only in the peripheral region but also in the facial nucleus in the brain stem [45], superior colliculus [46], and motor region of the spinal cord [47,48]. Antonucci et al. found cleaved SNAP-25 in the ipsilateral facial nucleus after a BTX-A injection into rat whisker muscles [45]. Matak et al. found that cleaved SNAP-25 fragments were present in the ventral horn and dorsal horn of the spinal cord after low-dose toxin injections into the gastrocnemius muscle and sciatic nerve [47]. These authors also identified that cleaved SNAP-25 was not detected in the spinal cord when they injected BTX-A into a sciatic nerve pretreated with colchicine, an axonal transport blocker. Therefore, they suggested that BTX-A showed a central effect by microtubule-dependent retrograde axonal transport [49]. Wang et al., however, cited the possibility that disassociated cleaved SNAP-25 might have migrated from the terminal to the cell body, suggesting that the discovery of cleaved SNAP-25 in the CNS does not necessarily reflect the activity of BTX-A in the CNS [50].

Retrograde axonal transport is well known as a transport pathway for various substances, such as tetanus toxin, and this fact suggests that BTX may also use the same pathway. Several studies have reported that the heavy chain, or the entire toxin, undergoes retrograde transport after a peripheral injection of BTX-A. Restani et al. directly monitored the endocytosis and axonal transport of BTX-A, and they showed that BTX-A was internalized by spinal cord motor neurons and underwent fast axonal retrograde transport [51]. Wang et al. reported that fluorescently labeled BTX heavy chains were detected in spinal cord motor neurons after injection into the mouse hindlimb, which demonstrated retrograde transport of BTX [50].

Several studies of the behavioral effects of BTX-A have demonstrated the central antinociceptive effect of BTX-A. Bilateral pain associated with experimental diabetes [52], carrageenan-induced hyperalgesia, and paclitaxel-induced peripheral neuropathy [53] or acidic saline-induced mirror pain, [54] can be bilaterally reduced by the unilateral injection of BTX-A in rats. Bach-Rojecky et al. reported that mechanical and thermal hypersensitivity of the ipsilateral side, as well as the contralateral side, were decreased after subcutaneous unilateral BTX injection into the plantar surface of the hindlimb [52]. In addition, Favre-Guilmard et al. reported a significant anti-hyperalgesic effect in the uninjected contralateral hindpaw after subcutaneous administration of BTX-A to the plantar surface in carrageenan-induced hyperalgesia and paclitaxel-induced peripheral neuropathy models. These results suggest that the antinociceptive effect of BTX-A cannot be explained by the peripheral action and it is possible that BTX-A has a central action through the retrograde axonal transport [53]. This process is also expected to be a major mechanism in the BTX-A action on central neuropathic pain.

## 4. Clinical Studies of BTX for Central Neuropathic Pain

### 4.1. Neuropathic Pain after Spinal Cord Injury

Two case series of clinical reports with very small sample sizes have evaluated the effect of BTX-A on neuropathic pain in patients with SCI. Jabbari et al. [55] reported cases of two patients with burning pain in a dermatome due to spinal cord lesion at the cervical level (tumor or stroke). BTX-A (OnabotulinumtoxinA) was injected subcutaneously at multiple points in the area of the burning pain and allodynia. The effect was assessed by the visual analogue scale (VAS) and clinical changes. One patient received 100 units of BTX-A. One week after the injection, the VAS score decreased from 8–10 to 2–3 points, and the frequency of severe spontaneous pain was reduced by 80%. The second patient received 80 units; skin sensitivity and spontaneous burning pain were significantly reduced after approximately 10 days, and this effect lasted approximately three months. Han et al. [56] reported a case of a patient with allodynia and dysesthesia of the lower limb. BTX-A was injected subcutaneously at ~10 units into the painful foot area and the effect was evaluated by the change in VAS score. After four weeks, the pain severity and burst frequency were reduced.

A recent study has been reported on the effect of BTX-A on SCI-associated neuropathic pain. Han et al. [57] reported the effects of BTX-A in a randomized, double-blind, and placebo-controlled study in 40 patients with SCI-associated neuropathic pain. Patients were treated with subcutaneous injections of BTX-A (200 units) or normal saline and the VAS score, the Korean version of the short-form McGill Pain Questionnaire, and the WHOQoL-BREF quality of life assessment were assessed at four and eight weeks. Thus, BTX-A has been shown to be effective in treating intractable chronic neuropathic pain in patients with SCI. The above studies are summarized in Table 1.

### 4.2. Post-Stroke Shoulder Pain

Central post-stroke pain occurs after a cerebrovascular event, including lesion of the brainstem, thalamus, and cerebral cortex, and may affect half of the body [73]. Several authors have described central post-stroke pain as a central neuropathic pain syndrome that can occur after a stroke in the body part that corresponds to the cerebrovascular lesion and is characterized by pain and sensory abnormalities, where other causes of obvious nociceptive, psychogenic, or peripheral neuropathic origin have been ruled out [74,75].

Post-stroke shoulder pain is a common disease with an incidence rate ranging from 21–72% [76,77]. Many studies have examined the effects of BTX on the treatment of post-stroke shoulder pain, but the results are conflicting, and, thus, drawing conclusions remains difficult.

Yelnik et al. [58] conducted a double-blind RCT of the effect of BTX on post-stroke shoulder pain in 20 patients. Ten patients were injected with 500 units of BTX-A (AbobotulinumtoxinA) in the subscapularis muscle, and 10 patients in the control group underwent a placebo injection in the same muscle. All participants underwent rehabilitation, including stretching exercises. Pain was improved in the BTX injection group at one week, and pain scores using a 10-point verbal scale at four weeks showed a significant difference between the two groups. Marco et al. [59] reported a double-blind RCT for evaluating the effect of BTX. In 14 patients, 500 units of BTX-A (AbobotulinumtoxinA) was injected into the pectoralis major muscles, and 15 patients in the control group were injected with a placebo. Transcutaneous electrical nerve stimulation was applied for six weeks. After one week, pain during shoulder movement decreased in both groups, but the VAS score in the BTX injection group decreased more significantly, and this trend continued until six months. However, no significant difference in the shoulder range of motion or spasticity was found between the two groups. Kong et al. [60] conducted a double-blind RCT of 17 patients. Five hundred units of BTX-A (AbobotulinumtoxinA) was injected into the pectoralis major and biceps brachii muscles in the experimental group and normal saline was injected into the same region in the control group. The VAS scores at 4, 8, and 12 weeks after injection were not significantly different between the two groups. The median baseline VAS score of the patients was 6, and the scores decreased by 2–3 points in both groups.

Lim et al. [61] reported a double-blind RCT of 29 patients. In the experimental group, 100 units of BTX-A (OnabotulinumtoxinA) was injected into the infraspinatus, pectoralis, and subscapularis muscles, along with intra-articular saline. In contrast, the control group received an intra-articular triamcinolone (40 mg) injection and saline was injected into the muscles described above. The numeric rating scale at 12 weeks was reduced by 4.2 ± 0.4 points in the BTX-A intramuscular injection group and by 2.5 ± 0.8 points in the intra-articular triamcinolone injection group. Intramuscular injection of BTX-A was superior to intra-articular injection of triamcinolone (*p* = 0.051). Boer et al. [62] conducted a double-blind RCT of 22 patients. They injected 100 units of BTX-A (OnabotulinumtoxinA) into the subscapularis muscle in the experimental group and injected saline in the control group. Vertical VAS scores were not significantly different between the two groups at 6 weeks and 12 weeks. Shaw et al. [63] reported the effects of BTX-A (AbobotulinumtoxinA) on spasticity, function, and pain in patients with spasticity of the upper limb after stroke. This study was a multicenter RCT called BoTULS. The pain rating and pain scale evaluated at one and three months showed no significant difference between the two groups, but the pain rating evaluated at 12 months showed a significant decrease in the injection group. Marciniak et al. [64] evaluated the effects of BTX by injecting 140–200 units of BTX-A (OnabotulinumtoxinA) or saline into a pectoralis major muscle, with or without a teres major muscle. At four weeks, worst pain ratings decreased in both groups, but no significant difference was found between the two groups. The above studies are summarized in Table 1.

Post-stroke shoulder pain is thought to be caused by multiple factors, including both the nervous system and mechanical factors. Post-stroke shoulder pain may be associated with spasticity, but it is difficult to determine whether spasticity acts as a mechanism to cause post-stroke shoulder pain, whether it is increased by the shoulder pain, or both. It is well known that the improvement of spasticity may be associated with an improvement of pain, but the correlation between spasticity and pain is not linear, and multiple factors may be involved [78]. Several studies have suggested a musculoskeletal origin for post-stroke shoulder pain. Musculoskeletal conditions, such as subluxation, tendinitis, adhesive capsulitis, rotator cuff tear, and subacromial bursitis, may contribute to post-stroke shoulder pain. Whether these diseases can cause post-stroke shoulder pain is controversial, because these conditions may result from stroke.

In a study by Zeilig [79], those with post-stroke shoulder pain had higher heat-pain thresholds and exhibited higher rates of hyperpathia, allodynia, and dysesthesia in the affected shoulder and leg than those without post-stroke shoulder pain. The authors suggested that the finding of altered thermal sensitivity, which indicates damage to the spinothalamic-thalamocortical tract, was not restricted to the shoulder, but rather characterized the affected side. An additional support to this central neuropathic pain proposition was the higher rate of damage to the parietal cortex in the post-stroke shoulder pain group. From these results, various factors appear to cause post-stroke shoulder pain, but it seems to be attributable to central neuropathic pain.

Two systematic reviews of the effects of BTX on shoulder pain, including post-stroke shoulder pain, have been reported. In a Cochrane report that focused on BTX-A for shoulder pain, the authors included six RCTs comparing BTX with a placebo or active treatment. Five RCTs in participants with post-stroke shoulder pain indicated that, compared with placebo, a single intramuscular injection of BTX-A significantly reduced pain at three to six months postinjection, but not at one month [80]. Another systematic review included nine RCTs of BTX injections in patients with shoulder pain. The shoulder pain was due to hemiplegia in six studies, adhesive capsulitis in one study, subacromial bursitis or shoulder impingement syndrome in one study, and arthritis in one study [81]. They concluded that BTX injection resulted in minor to moderate pain relief and an increase in shoulder abduction in patients with chronic shoulder pain. Based on these two reviews, BTX injection in post-stroke shoulder pain is expected to be effective in reducing pain.

### 4.3. Multiple Sclerosis

MS is a chronic disease in which focal demyelinating lesions of the CNS occur at multiple sites due to autoimmune inflammatory processes. The plaques, located in the subcortical, brainstem, or spinal cord, cause neurological symptoms and signs, including abnormal coordination, motor, sensory, and cognitive function. According to one report, approximately 65% of MS patients with spasticity are known to suffer from pain [82]. Pain appears in the form of central dysesthetic pain, trigeminal neuralgia, Lhermitte’s phenomenon, and tonic spasm. In a review published in 2013, the authors classified MS-related pain into nine categories and described each possible mechanism [82]. According to the authors, ongoing extremity pain is caused by thalamic or cortical deafferentation by multiple lesions along the spinothalamocortical pathways. In addition, Lhermitte’s phenomenon and painful tonic spasm are caused by demyelination of the dorsal column primary afferents and the corticospinal pathway, respectively, providing a possible mechanism for central neuropathic pain.

Various double-blind RCTs of BTX effects on MS-associated detrusor overactivity and spasticity are available. A preliminary report on the effects of BTX on spastic dysphagia, myokymia, tonic spasm, and internuclear ophthalmoplegia also exists. However, no RCT has been performed to evaluate whether BTX is effective for MS-related pain. In a recent prospective, open-label study of 131 patients with spasticity, 19% were patients with MS, and 60% reported a significant reduction in spasticity-related pain after BTX-A treatment [83]. MS-related pain has the characteristics of central neuropathic pain and conducting well-designed research on whether BTX is effective for MS-related pain will be necessary in the future.

### 4.4. Complex Regional Pain Syndrome

Complex regional pain syndrome (CRPS) is a painful disease that can result from an imbalance due to trauma. Unlike other neuropathic pain syndromes, CRPS is accompanied by additional signs, such as abnormal blood flow control, sweating, and active and passive motor impairment. Not all CRPS signs can be fully explained by the peripheral mechanism and several studies suggest there might be a central mechanism [84]. The CNS undergoes functional and structural changes in people with chronic pain and these changes are thought to be particularly important in CRPS because they cause central sensitization [85,86]. CRPS is often spread beyond the original injury and in many cases, it has been reported to spread to the contralateral extremity in a mirror pattern [87,88]. Regarding the bilateral spreading of CRPS signs, it was suggested that trans-synaptic changes in the spinal cord dorsal horn, contralateral to the affected side, may underlie this spreading [89]. It is unclear whether CNS alterations are a primary abnormality in the disease or a secondary change due to pain, but changes in the CNS play an important role in CRPS, thus, this review addresses the disease.

Carroll et al. prospectively investigated 18 subjects with CRPS [66]. Lumbar sympathetic blocks were accomplished with bupivacaine alone or an additional 75 units of BTX-A. The rate of pain return was significantly lower and the duration of pain reduction was longer in the group with BTX-A injection compared to that with local anesthetic alone. Safarpour et al. conducted a double-blind, randomized, controlled, and open-label extension study [67]. BTX-A was injected intradermally and subcutaneously into the allodynic area, but no significant response occurred after treatment. Kharkar et al. injected 10–20 units of BTX-A per muscle in 37 patients with CRPS-related spasm or dystonia in the neck and/or upper limb girdle [68] and found a statistically significant decrease in local pain scores compared with baseline. Several additional case reports and case series with very small sample sizes are listed in Table 1.

Although no systematic review of the effect of BTX on CRPS is available, most studies have shown BTX to be effective in reducing pain. CRPS also has a multifactorial mechanism, but as central sensitization is reported as a major mechanism, BTX is expected to effectively reduce the pain.

## 5. Conclusions

The mechanism of central neuropathic pain has been examined according to various hypotheses, including neuronal hyperexcitability and dysfunction of the spinothalamic tract. To date, various preclinical and clinical studies have been published on whether BTX may be effective for central neuropathic pain. BTX inhibits the secretion of substance P and CGRP from DRG, inhibits the expression of TRPV1 and P_2_X_3_, and induces a central effect through retrograde axonal transport. In addition, several studies have demonstrated its effect on central neuropathic pain associated with SCI, stroke, MS, and CRPS. Effects of BTX on neuropathic pain after SCI, post-stroke shoulder pain, and CRPS has been shown; therefore, it can be considered as one of the treatment options. In the future, well-designed studies will be necessary to assess the effects of BTX on central neuropathic pain, and, furthermore, effective injection sites, injection techniques, and adequate doses should be considered.

## Figures and Tables

**Table 1:** Summary of studies of botulinum toxin (BTX) for central neuropathic pain.

Author, Year	Study Design	Sample Size (*N*)	Diagnosis	Injection Site/Dose	Follow up	Pain Measure	Results
Jabbari, 2003 [55]	Case series	2	SCI	Subcutaneous injection at the site of allodynia/BTX-A 16–20 U/site		VAS	Pain was decreased; frequency of severe spontaneous pain was reduced
Han, 2014 [56]	Case report	1	SCI	Subcutaneous injection in the painful foot/BTX-A	Week 4	VAS	Pain severity and the frequency of burst was reduced
Han, 2016 [57]	Double-blind, randomized controlled study	40	SCI	Subcutaneous injection/BTX-A 200 U	Week 4, 8	VAS (100 mm), McGill Pain Questionnaire	Pain was reduced significantly in BTX-A treated group
Yelnik, 2007 [58]	Double-blind, randomized controlled study	20	stroke	Subscapularis muscle/BTX-A 500 U/injection + physical therapy	Week 1, 2, 4	verbal scale (10 point)	Pain improvement with BTX-A from first week
Marco, 2007 [59]	Double-blind, randomized controlled study	31	stroke	Pectoralis major muscle/BTX-A 500 U/injection + TENS for 6 weeks	Week 1, 4, 12, 24	VAS (100 mm)	Significantly greater pain improvement from the first week in BTX group
Kong, 2007 [60]	Double-blind, randomized controlled study	17	stroke	Pectoralis major, biceps brachii muscles/BTX-A 500 U	Week 4, 8, 12	VAS (0–10)	No difference in shoulder pain
Lim, 2008 [61]	Double-blind, randomized controlled study	29	stroke	Infraspinatus, pectoralis and subscapularis muscles + IA saline injection; IA triamcinolone (40 mg) injection + saline to the same muscles/BTX-A 100 U	Week 2, 6, 12	NRS	Significantly greater pain improvement in the BTX-A–treated at 12 weeks
Boer, 2008 [62]	Double-blind, randomized controlled study	22	stroke	Subscapular muscle/BTX-A 50 U, twice	Week 6, 12	VAS (vertical 100 mm)	No significant changes in pain
Shaw, 2011 [63]	Double-blind, randomized controlled study	333	stroke	Elbow, wrist and finger flexor muscles/ BTX-A, 4 times/injection + physical therapy 4 weeks	Week 4, 12, 48	verbal scale, NRS	Significant decrease at 12 months in the BTX group
Castiglione, 2011 [8]	Pilot study	5	stroke	IA shoulder joint/BTX-A 500 or 100 units	Week 2, 8	VAS	Decreased pain at 2 and 8 weeks after BTX-A injection
Marciniak, 2012 [64]	Double-blind, randomized controlled study	21	stroke	Pectoralis major ± teres major muscles/BTX-A 140–200 units	Week 2, 4, 12	VAS	Decreased pain scores at 4 weeks
Choi, 2016 [65]	Retrospective, unblinded, uncontrolled study	6	stroke	Subscapularis muscle/BTX-A	Week 1, 2, 4, 8	PI-NRS	Pain improvement with BTX-A injection
Carroll, 2009 [66]	Double-blind, randomized controlled study	18	CRPS	LSB/Bupivacaine 0.5% + 75 units of BTX-A	Week 4	VAS (10 cm)	The rate of pain return was significantly lower after LSB with BTA
Safarpour, 2010 [67]	Double-blind, randomized controlled study Uncontrolled, unblinded, open-label study	14 (6 control)	CRPS	Intradermally and subcutaneously into the allodynic area/ 5 units per site (total 40–200 units)	Week 3, 8	Brief pain inventory, PIQ, McGill pain questionnaire, QST, patients satisfaction scale	No significant response after injection; study terminated prematurely because of intolerance
Kharkar, 2011 [68]	Retrospective, unblinded, uncontolled study	37	CRPS	Upper limb girdle muscles/BTX-A 10–20 units per muscle (total 100 units)	Week 4	Likert scale (11 point)	43% decrease in local pain scores
Safarpour, 2010 [69]	Case series	2	CRPS	Trigger point in the proximal muscle/BTX-A 20 units per site	□	VAS (1–10)	Alleviate both myofascial pain syndrome and the distal allodynia, discoloration and, tissue swelling
Birthi, 2012 [70]	Case report	1	CRPS	Subcutaneous injection on the dorsum of the hand/BTX-A 5 units per site (total 100 units)	weekly, 12 weeks	McGill Pain Questionnaire	Able to decrease daily opioid medication by 20% at 8th week; pain returned to baseline at 12th week
Choi, 2015 [71]	Case series	2	CRPS	Lumbar sympathetic block/levovupivacaine 0.25% + 5000 units of BTX-B	Week 8	VAS, LANSS	Pain intensity and LANSS score were significantly reduced
Buonocore, 2017 [72]	Case report	1	CRPS	TP, FDL, FHL muscles, tibial nerve around the tarsal tunnel/BTX-A 120 units per muscle, twice	Week 36	□	Significant decrease in the frequency of acute dysesthesias

SCI: Spinal cord injury; CRPS: Complex regional pain syndrome; VAS: Visual analog scale; NRS: Numeric rating scale; IA: Intra-articular; LANSS: Leeds assessment of neuropathic symptoms and signs; LSB: Lumbar sympathetic block; TP: Tibialis posterior; FDL: Flexor digitorum longus; FHL: Flexor hallucis longus.
